# Genotoxicity of Novel Pyrazolo[4,3-*e*]tetrazolo[1,5-*b*][1,2,4]triazine Sulfonamides in Normal and Cancer Cells In Vitro

**DOI:** 10.3390/ijms24044053

**Published:** 2023-02-17

**Authors:** Mateusz Kciuk, Somdutt Mujwar, Beata Marciniak, Adrianna Gielecińska, Karol Bukowski, Mariusz Mojzych, Renata Kontek

**Affiliations:** 1 Department of Molecular Biotechnology and Genetics, University of Lodz, Banacha 12/16, 90-237 Lodz, Poland; 2 Doctoral School of Exact and Natural Sciences, University of Lodz, Banacha Street 12/16, 90-237 Lodz, Poland; 3 Chitkara College of Pharmacy, Chitkara University, Rajpura 140401, Punjab, India; 4 Department of Chemistry, Siedlce University of Natural Sciences and Humanities, 3 Maja 54, 08-110 Siedlce, Poland

**Keywords:** comet assay, genotoxicity, pyrazolo[4,3-*e*]tetrazolo[4,5-*b*][1,2,4]triazine, sulfonamides

## Abstract

Pyrazolo[4,3-*e*]tetrazolo[1,5-*b*][1,2,4]triazine sulfonamides constitute a novel group of heterocyclic compounds with broad biological activities including anticancer properties. The compounds investigated in this study (**MM134**, **-6**, **-7**, and **9**) were found to have antiproliferative activity against BxPC-3 and PC-3 cancer cell lines in micromolar concentrations (IC_50_ 0.11–0.33 µM). Here, we studied the genotoxic potential of the tested compounds with alkaline and neutral comet assays, accompanied by immunocytochemical detection of phosphorylated γH2AX. We found that pyrazolo[4,3-*e*]tetrazolo[1,5-*b*][1,2,4]triazine sulfonamides induce significant levels of DNA damage in BxPC-3 and PC-3 cells without causing genotoxic effects in normal human lung fibroblasts (WI-38) when used in their respective IC_50_ concentrations (except for **MM134**) and showed a dose-dependent increase in DNA damage following 24 h incubation of tested cancer cells with these agents. Furthermore, the influence of **MM** compounds on DNA damage response (DDR) factors was assessed using molecular docking and molecular dynamics simulation.

## 1. Introduction

DNA-damaging agents are frequently used in oncology to treat both hematological and solid malignancies. Platinum compounds (cisplatin, oxaliplatin, and carboplatin), cyclophosphamide, chlorambucil, and temozolomide are some of the most frequently utilized agents in cancer treatment. The above-mentioned drugs trigger apoptosis in cancer cells by altering the chemical structure of DNA. Unfortunately, the efficacy of these drugs can be greatly diminished by a variety of factors that contribute to drug resistance development. When drug efflux and/or metabolism rise, the intracellular concentration of an anticancer agent can be reduced, which impairs its capacity to inflict sufficient DNA damage and cell death [1,2].

Many of the cytotoxic drugs that are routinely used to treat cancer induce significant levels of DNA damage, which causes cell cycle checkpoint activation and results in cell cycle arrest and/or cell death [3]. However, the activity of anticancer drugs is not sufficiently selective, and damage to normal cells and tissues can occur. This may result in a variety of undesirable side effects [4]. Significant research effort has been devoted to the development of protein kinase inhibitors as therapeutic targets for the treatment of many human disorders, including diabetes, cancer, and hypertension. To date, Gleevec is one of the most prominent examples of an agent that targets an ABL protein kinase involved in several types of malignancies. The purinome consists of approximately 2000 distinct proteins expressed by the genome that utilize purines as substrates or as co-factors in the form of NAD, NADP, and co-enzyme A. The structures of representative gene family members within the purinome have revealed that these proteins bind purines in the same orientations as those reported in all protein kinases. The purinome is a rich pool of therapeutic targets. However, it also contains a wide collection of different proteins whose suppression may have unintended side effects [5].

A growing research area of heterocyclic compounds is still in the early stages of development, but it holds great promise for the discovery of new drugs. The development of new synthesis methods and the use of modern organic chemistry techniques have resulted in the rapid expansion of this area [6,7]. One of the primary goals of heterocyclic compound chemistry is the discovery of anticancer drugs, and one of the primers in organic anticancer chemistry is the synthesis of anticancer agents that structurally resemble the substrates of naturally occurring metabolic processes. Cell proliferation is slowed and apoptotic cell death is triggered when these critical mechanisms are interfered with [8,9,10]. Undoubtedly, the 1,2,4-triazines fused with five-membered heterocycles, which have exhibited bioisosteric resemblance to the purine core, are one of the most promising classes of agents with significant biological activity [11,12].

The pyrazolo[4,3-*e*][1,2,4]triazine class of compounds encompasses many unique chemical structures with broad biological activity, including inhibitory activity against carbonic anhydrase (CAs) [13,14], tyrosinase [15,16], urease [16], and anticancer activity as a result of their capacity to inhibit protein kinases such as cyclin-dependent kinases (CDKs) [17,18].

**Figure 1:**
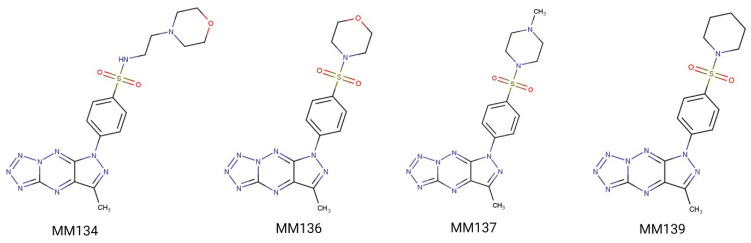
Investigated **MM** compounds: chemical structure of the four investigated sulfonamides **MM134**, **MM136**, **MM137**, and **MM139**.

**Figure 2:**
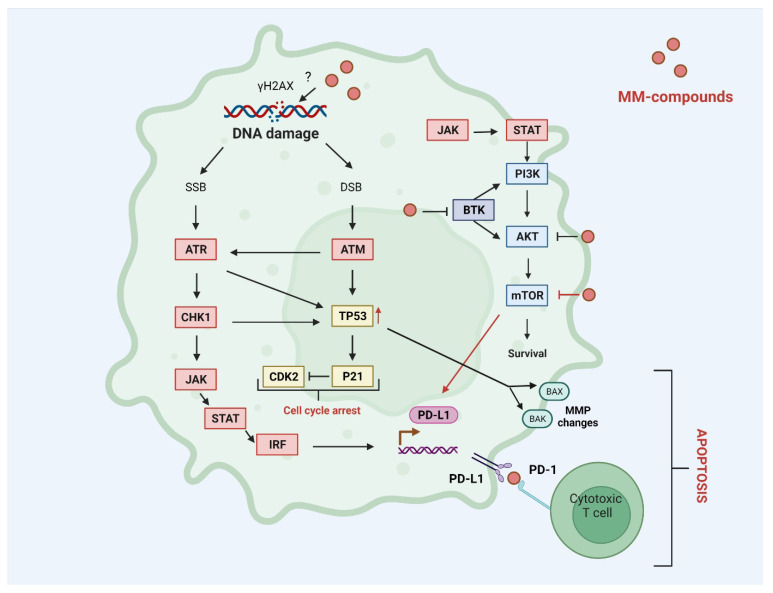
Predicted mechanism of action of **MM** compounds: **MM** compounds may act as BTK, PI3K-AKT-mTOR, and PD-L1 inhibitors that reduce cell survival and trigger apoptosis in a cytotoxic T-cell-dependent mechanism. Inhibition of mTOR kinase leads to up-regulation of PD-L1 expression. Increased expression of PD-L1 may result from the accumulation of DNA damage and activation of ATM/ATR/CHK1 and JAK/STAT/IRF signaling pathways. DNA damage leads to the up-regulation of TP53 tumor suppressor and induction of pro-apoptotic proteins including BAK and BAX involved in the intrinsic apoptosis pathway associated with MMP changes and increased production of P21 that works as an inhibitor of CDK2. AKT—RAC-alpha serine/threonine-protein kinase; ATM/ATR—serine-protein kinase ATM/ATR; BAX/BAK—pro-apoptotic protein BAX/BAK; BTK—tyrosine-protein kinase BTK; CDK2—cyclin-dependent kinase 2; CHK1—serine/threonine-protein kinase CHK1; DSB—double-strand break; IRF1—interferon regulatory factor 1; JAK—tyrosine-protein kinase JAK; MMP—mitochondrial membrane potential; mTOR—serine/threonine-protein kinase mTOR; PI3K—phosphatidylinositol-4,5-bisphosphate 3-kinase; PD-L1—programmed cell death 1 ligand 1; RTK—receptor tyrosine kinase; STAT—signal transducer and activator of transcription; TP53—cellular tumor antigen p53; γH2AX—phosphorylated histone protein H2AX.

Previously we have shown that the pyrazolo[4,3-*e*]tetrazolo[1,5-*b*][1,2,4]triazine sulfonamides (**MM134**, **MM136**, **MM137**, and **MM139**) (Figure 1) investigated in this study exhibited selective cytotoxic potential in pancreas adenocarcinoma (BxPC-3) and prostate cancer (PC-3) cell lines without causing a cytotoxic effect on human lung fibroblasts (WI-38). Moreover, **MM134**, **-6**, **-7**, and **-9** exhibited pro-apoptotic activity in BxPC-3 and PC-3 cells and are predicted to act through inhibition of AKT-mTOR and PD-1/PD-L1 pathways in cancer cells, as indicated by in silico results [19]. The proposed mechanism of action of pyrazolo[4,3-*e*]tetrazolo[1,5-*b*][1,2,4]triazine sulfonamides (**MM compounds**) is shown in Figure 2.

In this paper, we present the genotoxic activity of pyrazolo[4,3-*e*]tetrazolo[1,5-*b*][1,2,4]triazine derivatives (**MM134**, **-6**, **-7** and **-9**) examined with alkaline and neutral comet assays, accompanied by immunocytochemical detection of phosphorylated histone protein (γH2AX).

In addition, a trypan blue test was performed to assess the viability of tested cells, and the Alamar blue test to explore the metabolic activity of the cells after treatment with the tested compounds.

Molecular docking and molecular dynamic simulation studies were employed to assess the binding potential of tested compounds with target enzymes belonging to the DNA damage response (DDR) pathway.

## 2. Results

### 2.1. Trypan Blue Staining

**Figure 3:**
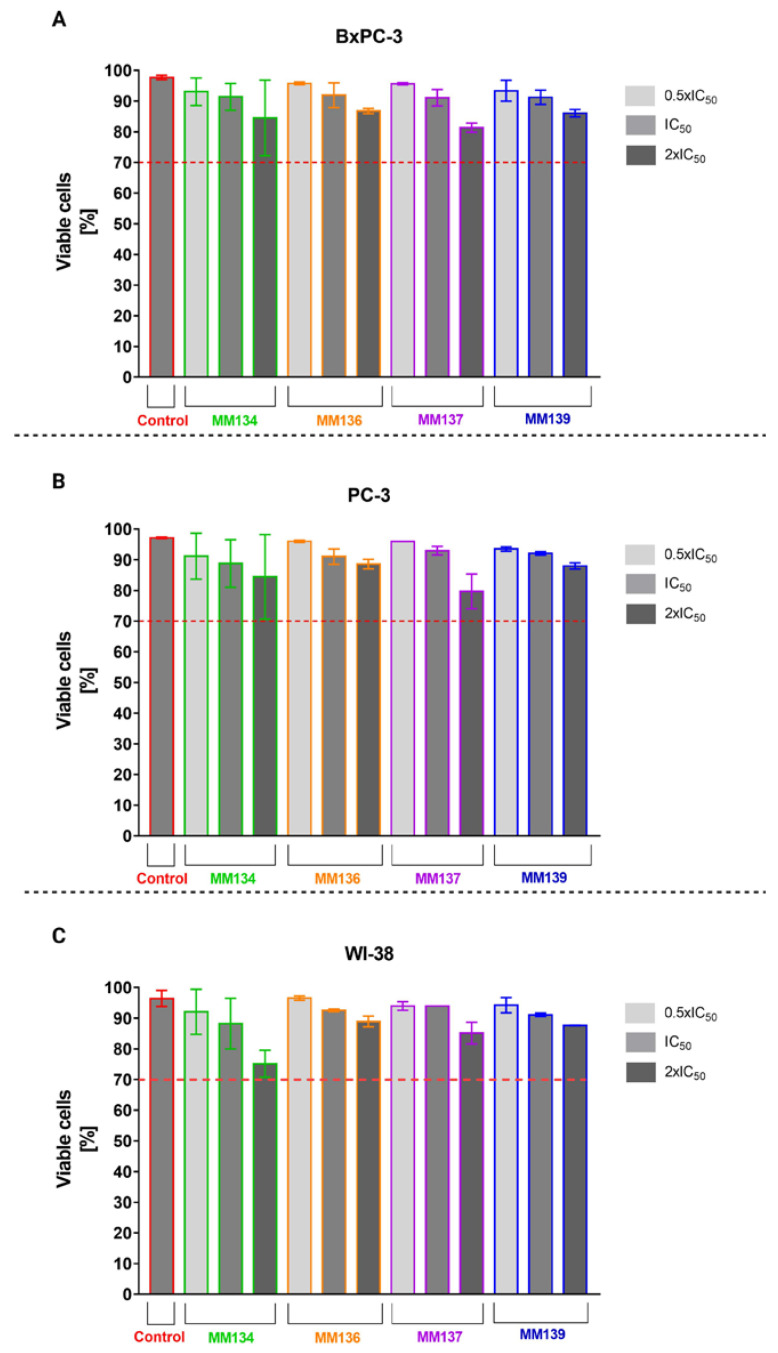
Trypan blue staining: effect of **MM134**, **-6**, **-7**, and **-9** used in concentrations of 0.5 (light gray), 1 (gray), and 2 (dark gray) times IC_50_ values on cancer cell (BxPC-3 (**A**) and PC-3 (**B**)) and normal human fibroblast (WI-38) (**C**) cell viability measured with trypan blue staining assay. Cells were exposed to tested compounds for 24 h at 37 °C. The mean values were obtained from three independent experiments. ±SD values.

Trypan blue staining used to determine cell viability in cancer cell lines (BxPC-3 (A), PC-3 (B)) and normal human fibroblasts (WI-38) (C) showed that all concentrations of the tested compounds **MM134**, **-6**, **-7**, and **-9** resulted in a minor decrease in cell viability following 24 h incubation at 37 °C; 5% CO_2_. The decrease in viability did not exceed the extreme value of 70% in any experimental series. However, **MM134** induced the highest reduction in cell viability in all tested cell lines (Figure 3).

### 2.2. Alamar Blue

**Figure 4:**
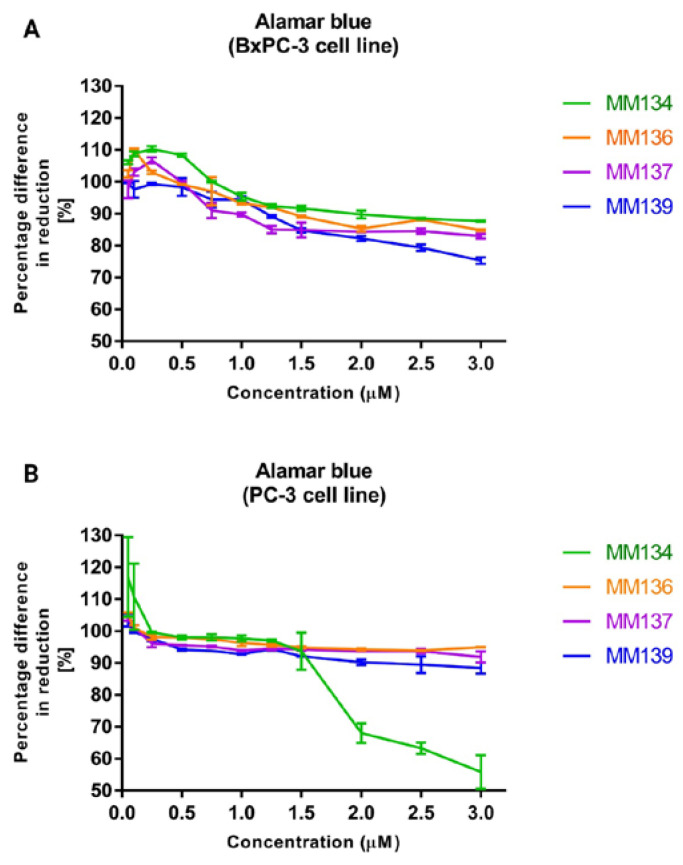
Alamar blue assay: the effect of **MM134**, **-6**, **-7**, and **-9** used in concentrations ranging from 0.05–3 µM on BxPC-3 (**A**) and PC-3 (**B**) cancer cells was measured with alamar blue assay. Cells were exposed to tested compounds for 24 h at 37 °C. The mean values were obtained from three independent experiments. ±SD values.

The alamar blue assay was used to confirm the results obtained with the trypan blue assay and was performed before the assessment of the compounds’ genotoxicity with the neutral comet assay. The results obtained from two independent experiments are presented in Figure 4.

Twenty-four hour incubation of BxPC-3 and PC-3 cells with tested compounds reduced the viability of cells up to the level of 75.3 ± 0.99% (**MM139**) in the BxPC-3 cell line, and 88.4 ± 1.72% (**MM139**) in the PC-3 cell line with the highest (3 µM) concentration used in the assay. Similarly to the trypan blue assay, tested compounds did not reduce cell viability to a point below 75% when used in concentrations ranging from 0.08–0.66 µM for the BxPC-3 cell line and 0.055–0.34 µM in the PC-3 cell line, where the concentration range represents the concentration values between the lowest 0.5 × IC_50_ value and highest 2 × IC_50_ value obtained for all tested compounds in a given cell line. Moreover, a higher reduction of cell viability was observed in the BxPC-3 cell line compared with the PC-3 cell line (except for the **MM134** compound). **MM139** derivative caused the highest decrease in cell viability in BxPC-3 cells (75.3 ± 0.99% for 3 µM concentration). In contrast, in the PC-3 cell line, the **MM134** compound induced a rapid decrease in cell viability in concentrations above 1.5 µM, reaching 55.8 ± 5.27% at the concentration of 3 µM.

### 2.3. Alkaline Comet Assay

#### 2.3.1. Cancer Cells

**Figure 5:**
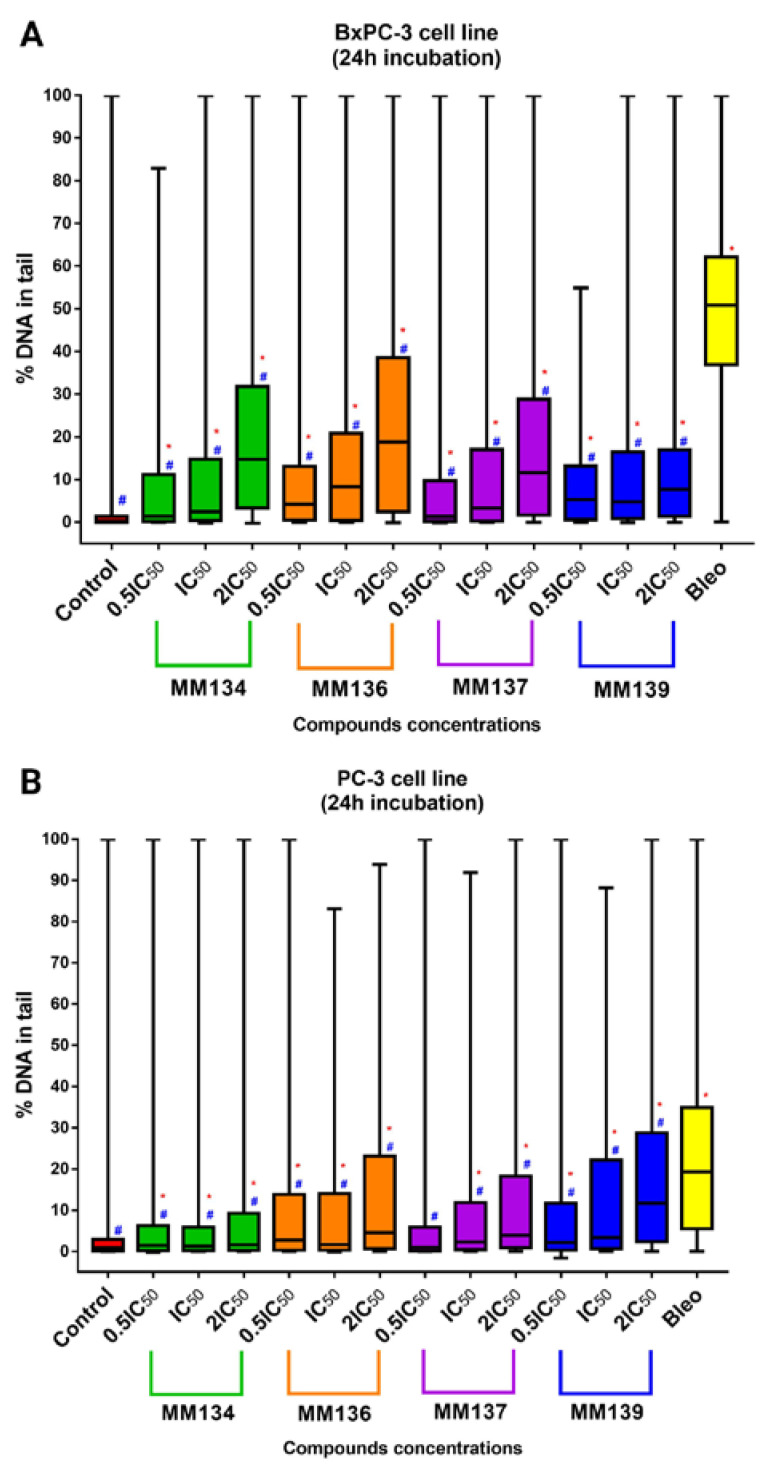
DNA damage in cancer cells estimated with 24 h alkaline comet assay: determination of DNA damage induced by 0.5 × IC_50_, IC_50_, and 2 × IC_50_ concentrations of **MM134**, **-6**, **-7**, and **-9** in cancer cell lines (BxPC-3 (**A**), PC-3 (**B**)). Data are represented as median tail DNA % with interquartile range and minimal and maximal values. The Kruskal–Wallis test was used to show a statistically significant difference between groups. Multiple comparisons using mean ranks for all groups module of Statistica software were used. In all groups, *N* > 200. * significant difference compared to the negative control; *p* < 0.05. # significant difference compared to the positive control (20 µM bleomycin); *p* < 0.05.

**Figure 6:**
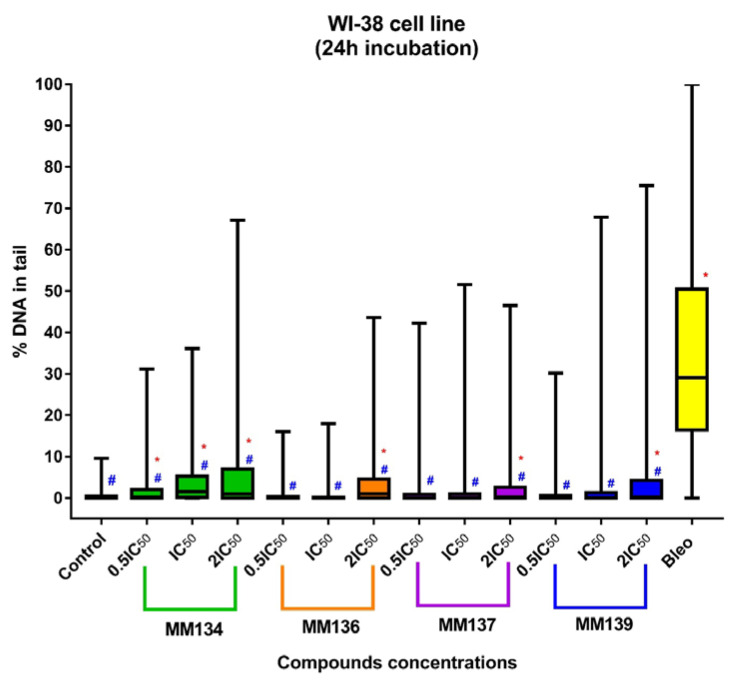
DNA damage in normal cells estimated with 24 h alkaline comet assay: determination of DNA damage induced by 0.5 × IC_50_, IC_50_, and 2 × IC_50_ concentrations of **MM134**, **-6**, **-7**, and **-9** in the WI-38 cell line. Data are represented as median tail DNA % with interquartile range and minimal and maximal values. The Kruskal–Wallis test was used to show a statistically significant difference between groups. Multiple comparisons using mean ranks for all groups module of Statistica software were used. In all groups, *N* > 200. * significant difference compared to the negative control; *p* < 0.05. # significant difference compared to the positive control (20 µM bleomycin); *p* < 0.05.

**Figure 7:**
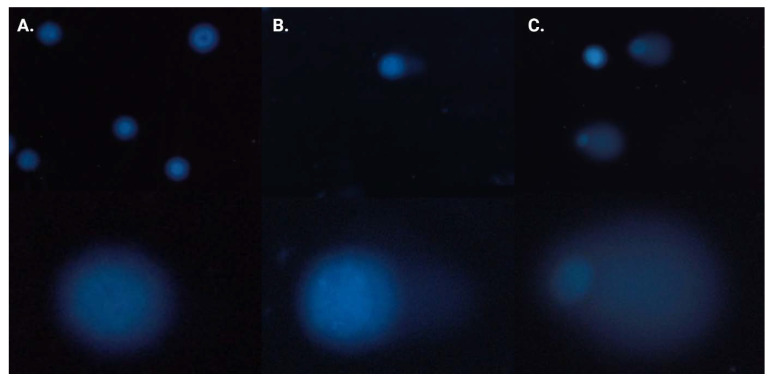
Alkaline comet assay results: images of comets were obtained in an alkaline comet assay for the PC-3 cell line: (**A**) control samples, (**B**) cells treated with 2 × IC_50_ (0.34 µM) of **MM139**, and (**C**) 20 µM bleomycin. Upper images are shown in magnification of 20×. Lower images are shown in arbitrary magnification.

DNA damage was assessed using an alkaline comet assay with the support of *CASP* software [20]. Data were presented as median tail DNA percent (%) with interquartile range and minimal and maximal values. Median tail DNA (%) following incubation of cells with three concentrations (0.5 × IC_50_, IC_50_, and 2 × IC_50_) of **MM134**, **-6**, **-7**, and **-9** were compared to negative (estimated DNA damage for BxPC-3 cells: median = 0.18%; mean = 2.04%; and PC-3 cells: median = 0.9%; mean = 3.33%) and positive control (20 µM of bleomycin) (estimated DNA damage for BxPC-3 cells: median = 50.8%; mean = 48.3%; and PC-3 cells: median = 19.31%; mean = 22.07%) as shown in Figure 5 (for the BxPC-3 cell line (A) and PC-3 cell line (B)) and in Figure 6 for the WI-38 cell line. An increase in DNA damage was observed following 24 h incubation with the tested compounds that was not due to their cytotoxicity as determined by the trypan blue viability test. Examples of comet images obtained in an alkaline comet assay for the PC-3 cell line are shown in Figure 7.

Tested compounds used in concentrations followed by IC_50_ values (0.5 × CI_50_, IC_50_, and 2 × IC_50_) induced a dose-dependent and statistically significant increase in DNA damage compared with the negative and positive control (*p* < 0.05) in both BxPC-3 and PC-3 cell line (except **MM137** used in 0.5 × IC_50_ compared to negative control in PC-3 cell line) following 24 h incubation time. DNA damage expressed as a median of %“tail DNA %” parameter obtained in CASP software for cells incubated with IC_50_ concentrations of tested pyrazolo[4,3-*e*]tetrazolo[1,5-*b*][1,2,4]triazine derivatives descendent in the order of: **MM136** (IC_50_ = 0.25 µM; median = 8.28%; mean = 14.36%), **MM139** (IC_50_ = 0.33 µM; median = 4.74%; mean = 10.85%), **MM137** (IC_50_ = 0.16 µM; median = 3.28%; mean = 12.07%) and **MM134** (IC_50_ = 0.32 µM; median = 2.5%; mean = 10.9%) for BxPC-3 cell line and **MM139** (IC_50_ = 0.17 µM; median = 3.35%; mean = 14.22%), **MM137** (IC_50_ = 0.11 µM; median = 2.32%; mean = 11.05%), **MM136** (IC_50_ = 0.13 µM; median = 1.63%; mean = 12.08%) and **MM134** (IC_50_ = 0.16 µM; median = 1.03%; mean = 7.37%) for PC-3 cell line (Figure 5).

#### 2.3.2. Normal Human Fibroblasts (WI-38 Cell Line)

In contrast, in the WI-38 cell line (Figure 6), **MM136**, **-7**, and **-9** induced statistically significant (*p* < 0.05) increases in DNA damage compared with negative (median = 0.05%; mean = 0.58%) and positive control (median = 29.13%; mean = 32.6%) only in 2 × IC_50_ concentrations. **MM134** induced a statistically significant increase in DNA damage compared with either negative or positive control. DNA damage was expressed as a median of the tail DNA % parameter following incubation of cells with tested pyrazolo[4,3-*e*]tetrazolo[1,5-*b*][1,2,4]triazine derivatives in IC_50_ concentrations descendent in the order of: **MM134** (IC_50_ = 0.65 µM; median = 1.5%; mean = 4.19%), **MM137** (IC_50_ = 0.27; µM; median = 0.09%; mean = 1.26%), **MM139** (IC_50_ = 0.54 µM; median = 0.085%; mean = 2.97%) and **MM136** (IC_50_ = 0.48 µM; median = 0.01%; mean = 0.72%). Once again, an increase in DNA damage levels was observed with an increase in the compound concentration used (dose–response relationship), which is characteristic of genotoxic compounds. However, in most cases, the increase was not statistically significant (except for **MM134** in all tested concentrations and **MM136**, **-7**, **-9** in 2 × IC_50_ concentrations). Of particular importance, the tested compounds induced lower levels of DNA damage in the normal cell line compared with cancer cell lines.

The difference between the genotoxic activity of the tested compounds and positive control was more profound in the BxPC-3 cell line compared with the PC-3 cell line, where derivatives induced levels of DNA damage more closely similar to that induced by bleomycin.

In summary, **MM134**, **-6**, **-7**, and **-9** exhibited genotoxic activity in BxPC-3 and PC-3 cancer cell lines. The tested compounds exhibited varied DNA-damaging capacities across the cell lines used in the study. DNA damage observed after 24 h incubation with the compounds in IC_50_ concentrations was highest in the BxPC-3 cell line when cells were exposed to **MM136** and **MM139** derivatives, while exposure to **MM139** and **MM137** resulted in the most profound induction of DNA damage in the PC-3 cell line. Furthermore, the BxPC-3 cell line exhibited higher sensitivity to both compounds and bleomycin used in the experiment as a positive control. In contrast, incubation of normal human fibroblasts (WI-38 cell line) with the tested compounds resulted in the induction of lower levels of DNA damage compared with the cancer cell lines. Moreover, a dose-dependent increase in DNA damage was observed after a 24 h incubation of cancer cells with increasing concentrations of the tested compounds (dose–response relationship), which is considered a hallmark of genotoxic compounds.

The treatment of cells with **MM** compounds resulted in a substantial heterogeneity of DNA damage, with values ranging from 0 to 100% tail DNA in the samples, which is consistent with the results obtained by other authors. This matter of producing 100% of tail DNA % can be attributed to some sort of bias using the software or the quality of the obtained images [21,22]. The occurrence of comets with >90% of DNA in the tail in the negative control samples could be attributed to the apoptosis of single cells and is a natural phenomenon, resulting, for example, from the preparation of samples. These comets are commonly referred to as “hedgehogs”. Hedgehogs are produced, for example, when cells are subjected to moderate exposure to hydrogen peroxide; however, they are no longer visible after the cells have been incubated for a short amount of time. This is not because the DNA has become even more damaged and disappeared from the gel; rather, it is because the DNA is being repaired. The comet assay has the potential to identify the first stages of apoptosis; however, as the process continues, the comets vanish, leaving behind a smear of unattached DNA. It is evident that hedgehogs can correspond to one level on a continuum of genotoxic damage, that they are not diagnostic of apoptosis, and that they should not be viewed as a sign of cytotoxicity [23].

### 2.4. Neutral Comet Assay

In contrast to the alkaline comet assay, which detects various types of DNA damage, including DNA double-strand breaks (DSBs), single-strand breaks (SSBs), and alkali-labile sites, the neutral comet assay is specific for the assessment of DSB induction [24].

**Figure 8:**
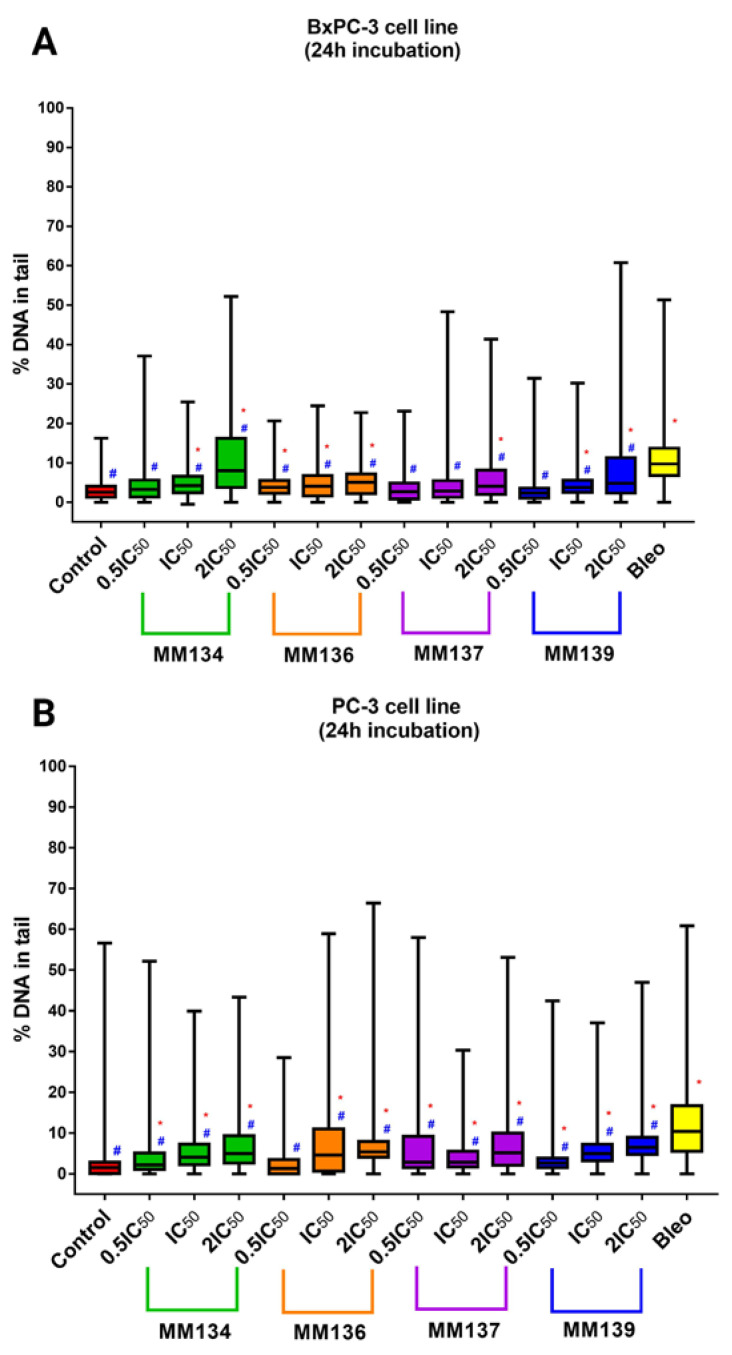
DNA damage in cancer cells estimated with 24 h neutral comet assay: determination of DNA damage (double-strand breaks) induced by 0.5 × IC_50_, IC_50_, and 2 × IC_50_ concentrations of **MM134**, **-6**, **-7**, and **-9** in cancer cell lines (BxPC-3 (**A**), PC-3 (**B**)) after 24 h incubation of cells with tested compounds. Data are represented as median tail DNA % with interquartile range and minimal and maximal values. The Kruskal–Wallis test was used to show a statistically significant difference between groups. Multiple comparisons using mean ranks for all groups module of Statistica software were used. In all groups, *N* > 200. * significant difference compared to the negative control; *p* < 0.05. # significant difference compared to the positive control (20 µM bleomycin); *p* < 0.05.

The neutral comet assay was performed analogously to the alkaline version of the method. Again, *CASP* software [20] was used to obtain median tail DNA percent (%) values with interquartile range and minimal and maximal values. Median tail DNA (%) following incubation of cancer cells (BxPC-3 (A) and PC-3 (B)) with three concentrations (0.5 × IC_50_, IC_50_, and 2 × IC_50_) of **MM134**, **-6**, **-7**, and **-9** was compared to a negative and positive control (20 µM of bleomycin) (Figure 8).

The tested compounds used in concentrations followed by IC_50_ values (0.5 × IC_50_, IC_50,_ and 2 × IC_50_) induced a dose-dependent and statistically significant increase in DNA damage compared with the negative and positive control (*p* < 0.05) in 2 × IC_50_ concentrations for both BxPC-3 and PC-3 cells following 24 h incubation time. A statistically significant increase in DNA damage compared to negative and positive control was also observed for cells incubated with IC_50_ concentrations of **MM134**, **-6**, and **-9** in the BxPC-3 cell line and for all compounds in the PC-3 cell line. DNA damage was expressed as a median of the tail DNA % parameter obtained in CASP software for cells incubated with IC_50_ concentrations of tested compounds descendent in the order of: **MM134** (IC_50_ = 0.32 µM; median = 4.21%; mean = 5.11%), **MM136** (IC_50_ = 0.25 µM; median = 4.10%; mean = 5.08%), and **MM139** (IC_50_ = 0.33 µM; median = 3.72%; mean = 4.86%) for the BxPC-3 cell line and **MM139** (IC_50_ = 0.17 µM; median = 5%; mean = 6.07%), **MM136** (IC_50_ = 0.13 µM; median = 4.6%; mean = 8%), **MM134** (IC_50_ = 0.16 µM; median = 4.09%; mean = 5.72%), and **MM137** (IC_50_ = 0.11 µM; median = 2.82%; mean = 4.45%) in the PC-3 cell line. DNA migration increased with the escalation of compound dose, which is characteristic of genotoxic compounds (dose–response relationship). All compounds induced statistically significant lower levels of DNA damage compared with 20 µM bleomycin used as the positive control (estimated DNA damage for BxPC-3 cells: median = 9.7%; mean = 11.15%; and PC-3 cells: median = 10.4%; mean = 12.9%).

**Figure 9:**
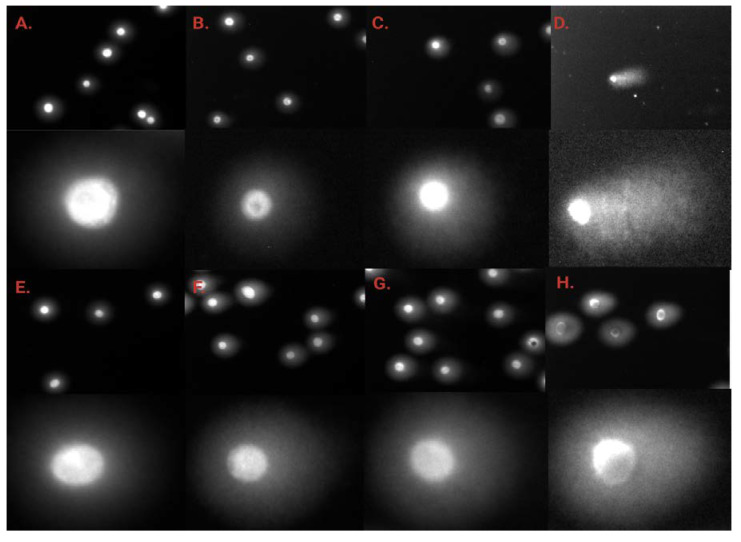
Neutral comet assay results: images of comets obtained in neutral comet assay for the BxPC-3 (**A**–**D**) and PC-3 (**E**–**H**) cells after incubation with **MM134** (**A**) control samples, (**B**) cells treated with 0.5 × IC_50_, (**C**) IC_50_ and (**D**) 2 × IC_50_ concentrations of the tested compound, and **MM139** (**E**) control samples, (**F**) cells treated with 0.5 × IC_50_, (**G**) IC_50_ and (**H**) 2 × IC_50_ concentrations of the tested compound. Upper images are shown in magnification of 20×. Lower images are shown in arbitrary magnification.

In summary, the tested compounds induced a statistically significant increase in DSB frequency in the BxPC-3 and PC-3 cell lines. **MM134** and **MM136** induced the highest levels of DSBs in the BxPC-3 cell line, while **MM139** and **MM136** induced more DSBs in PC-3 cells. For most of the compounds, an increase in DSB frequency was observed following incubation of cells with increasing concentrations of the compound. A dose–response relationship characteristic of genotoxic compounds was observed. Images of comets obtained in the neutral comet assay for the BxPC-3 and PC-3 cells after incubation with **MM134** and **MM139** are presented in Figure 9.

### 2.5. γH2AX Staining

**Figure 10:**
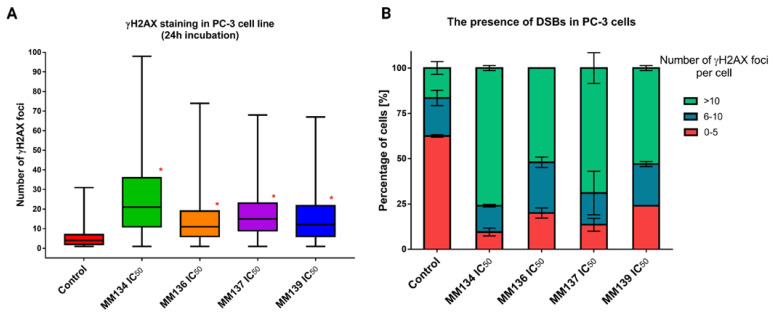
γH2AX staining results in PC-3 cells: determination of DNA damage (double-strand breaks—DSBs) induced by IC_50_ concentrations of **MM134**, **-6**, **-7**, and **-9** in PC-3 cancer cell line. (**A**) Data are represented as median γH2AX foci number per cell in each group with interquartile range and minimal and maximal values. The Kruskal–Wallis test was used to show a statistically significant difference between groups. Multiple comparisons using mean ranks for all groups module of Statistica software were used. In all groups, *N* > 200. * significant difference compared to the negative control (*p* < 0.05). (**B**) The graph presents the mean (±SD) percentage of cells that have a defined number of γH2AX foci in the sample.

To further examine DSB occurrence following 24 h incubation of cells with the tested compounds, a γH2AX staining assay was performed. γH2AX phosphorylation on serine 139 occurs following the occurrence of DNA damage and activation of a complex signaling network known as DDR. Therefore, γH2AX works as a sophisticated marker of DSBs [25]. The median numbers of γH2AX foci for PC-3 cells incubated with IC_50_ and 2 × IC_50_ concentrations of tested compounds were obtained using ImageJ software. PC-3 cells were used due to their large size, allowing an accurate measurement of foci quantity with good resolution. For BxPC-3 cells, we could not obtain the resolution necessary to quantify individual foci. Therefore, we decided to use only one cell line in the assay (Figure 10).

**Figure 11:**
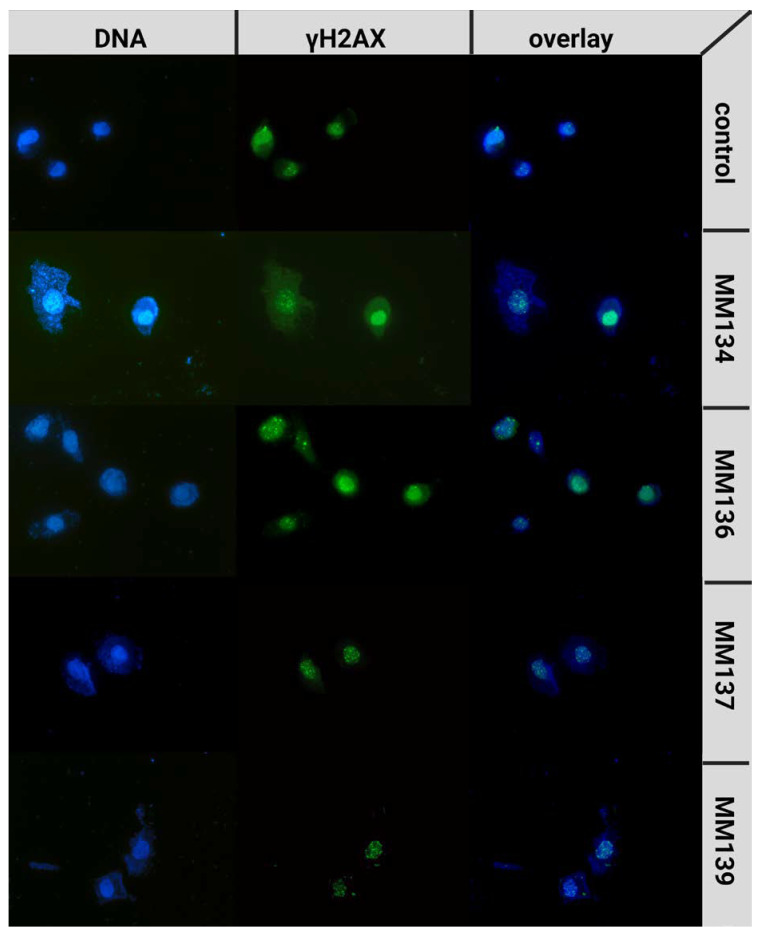
Examples of γ-H2AX stained cells: PC-3 cells were treated with **MM134**, **-6**, **-7**, and **-9** in concentrations concerning their IC_50_ values. Images are shown in magnification of 40×.

A statistically significant increase (*p* < 0.05) in γH2AX foci formation was observed after 24 h incubation with the tested compounds compared to a negative control (median = 4; mean = 5.68 γH2AX foci). For cells incubated with IC_50_ concentrations of the tested compounds, the median numbers of γH2AX foci were descendent in the order of: **MM134** (IC_50_ = 0.16 µM; median = 21; mean = 25.8), **MM137** (IC_50_ = 0.11 µM; median = 15; mean = 17.52), **MM139** (IC_50_ = 0.17 µM; median = 12; mean = 15.37) and **MM136** (IC_50_ = 0.13 µM; median = 11; mean = 14.71). Examples of anti-γH2AX stained cells are shown in Figure 11.

### 2.6. Computational Studies

#### 2.6.1. Molecular Docking

Molecular docking studies were performed to examine the binding modes and binding affinities of **MM** compounds with macromolecular targets from DDR. Moreover, the obtained results were compared with the binding properties evaluated for reference ligands that were co-crystalized with the macromolecular structures. The results are shown in Table 1. Conformations of MM compounds as well as native ligands with DDR targets were produced using Autodock and were clustered using the cluster module according to the root mean square deviation (RMSD) criterion of the same software. The clusters that were ranked to have the lowest binding energies (kcal/mol) were chosen for further investigations.

**Figure 12:**
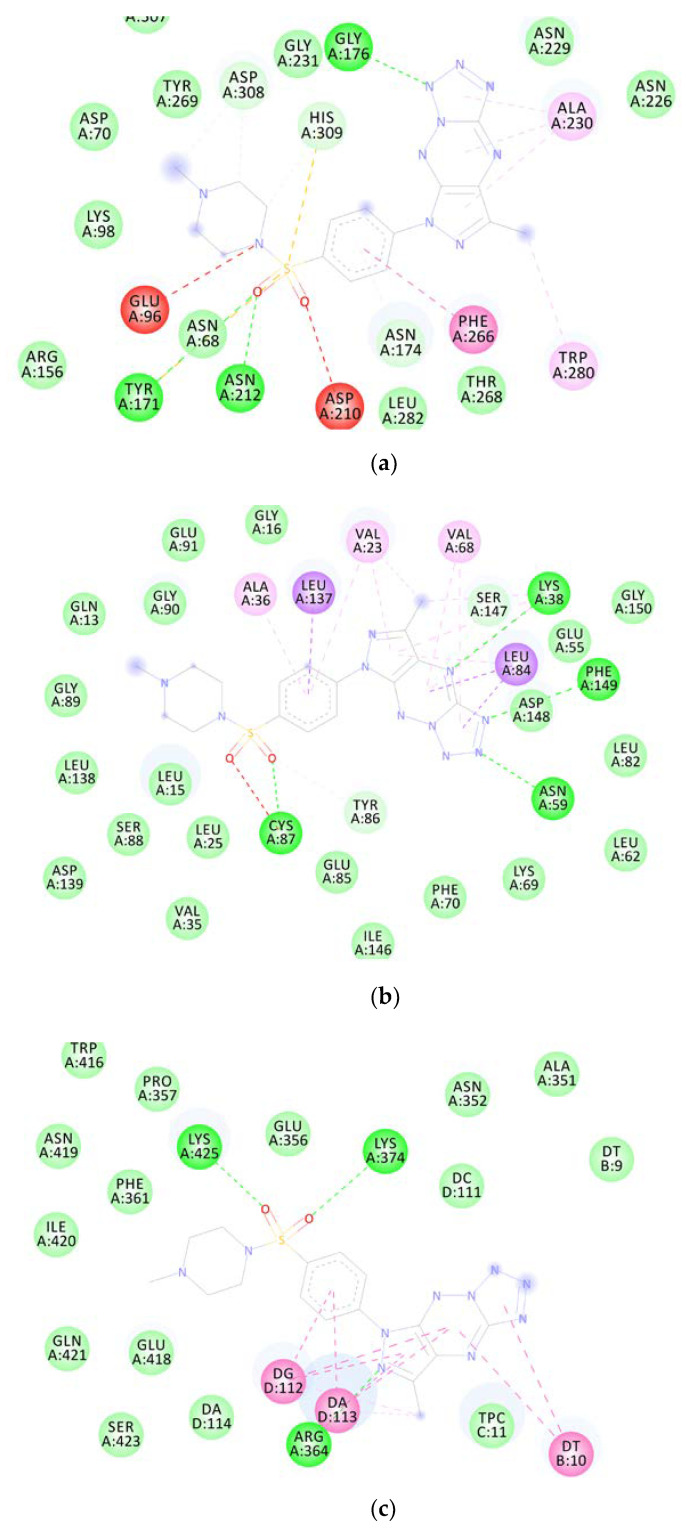
Graphical representation of molecular docking results: two-dimensional binding interactions of **MM137** with human APE1 (**a**), CHK1 (**b**), and TOPO1 (**c**).

Molecular docking studies revealed that **MM** compounds exhibited better binding affinities for APE1, CHK1, and TOPI macromolecular targets than the respective reference ligands because of the increased number of chemical interactions observed. Figure 12 depicts the two-dimensional binding interactions of **MM137** with APE1 (a), CHK1 (b), and TOPI (c), respectively.

#### 2.6.2. Molecular Dynamics

**Figure 13:**
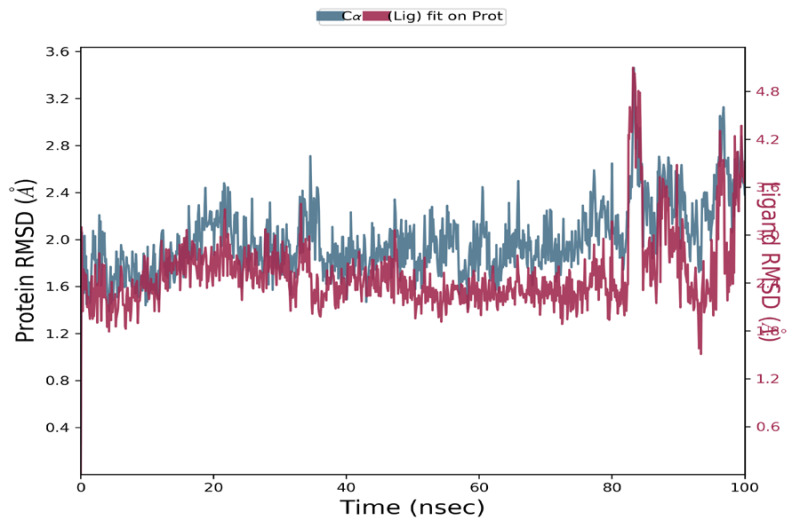
The RMSD plot for molecular dynamics simulation of **MM137** and CHK1: RMSD values of the ligand **MM137** complexed with CHK1 recorded during the MD simulation for 100 ns.

The macromolecular complexes of **MM137** against five target proteins—ATR, APE1, CHK1, TOPO1, and WEE1—exhibited the highest binding; however, the energy differences required for the competitive inhibition of target enzymes ATR and WEE1 were relatively small when compared with their reference ligands. Thus, the macromolecular complexes of **MM137** with APE1, CHK1, and TOPO1 were considered for executing simulation analysis. Molecular dynamics was used to assess the thermodynamic stability of **MM137** with APE1, CHK1, and TOPO1 by performing a molecular dynamic (MD) simulation of 100 ns using Schrodinger’s Desmond software. The molecular dynamics simulation of the three shortlisted macromolecular complexes of **MM137** with APE1, CHK1, and TOPO1 revealed that the macromolecular complex **MM137** with the CHK1 kinase was highly stabilized for the whole 100 ns of the simulation timeframe. RMSD analysis was performed. It was observed that the macromolecular backbone as well as the complex ligand maintained a stable conformation throughout the simulation period of 100 ns, with an RMSD value ranging within 1.6–2.8 Å for the macromolecular backbone and 2.4–4.8 Å for the ligand molecule (Figure 13). The protein–ligand interactions and contacts indicated the important amino acid residues that were crucial in providing stability to the complex. These important amino acid residues included Ala36, Leu84, Glu85, Val68, Lys38, Glu134, Asn135, Leu137, Val23, Cys87, Tyr86, and Leu15. During the simulation, the residues Cys87, Asn59, and Asp148 were directly involved in H-bonding. Therefore, it was concluded from the MD simulation studies that the H-bonding and water-linking interactions that were previously identified via Cys87, Asn59, and Asp148 residues are essential for stabilization of the protein–ligand complex.

**Figure 14:**
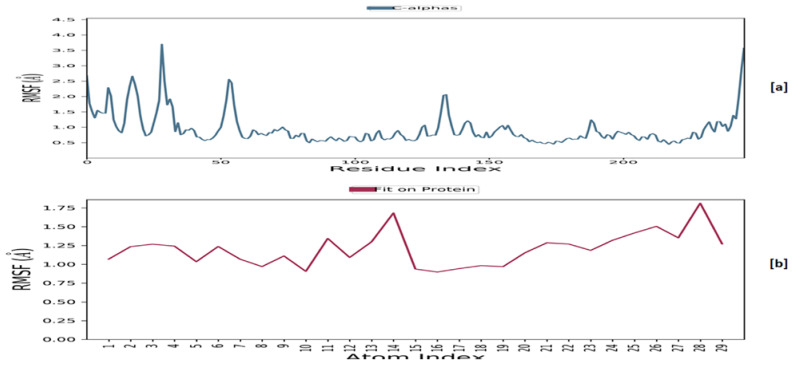
The RMSF plot for **MM137** and CHK1: RMSF for the **MM137** (**a**) and the macromolecular backbone of CHK1 (**b**) obtained after MD simulation.

It was discovered that the root-mean-square fluctuation (RMSF) (Figure 14), except for a few residues, remained within the range of 2.0 Å for the macromolecular backbone and 1.5 Å for the complexed ligand, indicating fluctuation within the acceptable range.

## 3. Discussion

Genotoxicity describes the property of a chemical that has a harmful effect on the genetic material of a cell (DNA and/or RNA), hence impairing the integrity of the cell genome. Genetic toxicology is an area of research that is concerned with the investigation of agents or substances that have the potential to cause damage to DNA and chromosomes. It should be noted that genotoxicity is frequently confused with mutagenicity. All mutagens are also genotoxic, but not all genotoxic compounds are mutagenic. The interaction of the genotoxic chemical with the DNA structure and sequence results in the destruction of genetic material [26,27]. Many well-established in vitro assays exist and have been successfully used to predict the genotoxicity of compounds. However, at this time, they cannot be considered a complete replacement for the animal experiments that are currently used to evaluate the safety of drugs [28]. Mitosis is a critical phase in the cellular response to genotoxic agents in human cells. Cells with damaged DNA recruit phosphorylated γH2AX, ATM, and ATR serine-protein kinases that phosphorylate checkpoint kinase 1 (CHK1), which in consecutive events triggers G2-phase cell cycle arrest or apoptosis [29].

More recently, a new compound known as **MM129** (pyrazolo[4,3-*e*]tetrazolo[1,5-*b*][1,2,4]triazine sulfonamide) has been demonstrated to effectively limit cell viability by inhibiting Bruton’s tyrosine kinase (BTK), which is involved in cell proliferation [30]. Furthermore, **MM129** exhibits anticancer efficacy in colon cancer xenograft mouse models. Not only does **MM129** have the potential to inhibit intracellular pathways that promote carcinogenesis, but it also has the potential to lower the protein levels of programmed death ligand 1 (PD-L1). It is known that PD-L1 is the primary ligand of programmed death 1 (PD-1), a coinhibitory receptor that can be expressed either constitutively or be induced in myeloid cells, lymphoid cells, normal epithelial cells, and cancer cells. PD-1/PD-L1 interaction is critical in the establishment of immunological tolerance under physiological conditions because it prevents excessive immune cell activity that can lead to tissue destruction and autoimmunity. The expression of PD-L1 is an immune evasion strategy used by a variety of cancers, and it is often associated with a much worse treatment prognosis [31,32]. Exposure of DLD-1 and HT-29 cells to **MM129** resulted in a decrease in the expression of RAC-alpha serine/threonine-protein kinase (AKT), serine/threonine-protein kinase (mTOR), and CDK2 [31]. Furthermore, incubation of colon cancer cells with **MM131**—another pyrazolo[4,3-*e*]tetrazolo[1,5-*b*][1,2,4]triazine sulfonamide—induced apoptosis of DLD-1 and HT-29 cells with observed down-regulation of mTOR kinase, soluble intercellular adhesion molecule-1 (sICAM-1), and cathepsin, together with up-regulation of beclin-1 B [33]. We have also shown, with the use of an alkaline/neutral version of the comet assay and γ-H2AX staining, that **MM129**, **MM130**, and **MM131** sulfonamides exhibited genotoxic activity in four investigated cancer cell lines (HeLa, HCT-116, PC-3, and BxPC-3) [34]. The occurrence of DNA damage following exposure to genotoxic agents would normally trigger the DDR responsible for the activation of DNA repair mechanisms or elimination of the severely damaged cell. It was also shown that via activation of cell cycle checkpoints, generation of neoantigen epitopes, and other mechanisms, DDR can regulate the expression of PD-L1 on tumor cells. Several investigations supported the evidence that the main DDR branch—the ATM/ATR/CHK1 pathway—may upregulate the expression of PD-L1 through the JAK-STAT1/3-IRF1 pathway (for full names of the proteins, see abbreviations section) [35,36]. Furthermore, inhibition of the mTOR kinase may increase the expression of PD-L1 on cancer cell surfaces. Higher levels of PD-L1 expressed by cancer cells may increase the availability of epitopes to which anti-PD-L1 agents can bind [37].

In our previous studies [19], **MM134**, **-6**, **-7**, and **-9** exhibited cytotoxicity against cancer cell lines including a pancreas adenocarcinoma cell line (BxPC-3), where they showed activity in the range of 0.16–0.33 µM, and prostate adenocarcinoma cell line (PC-3), where the activity ranged from 0.11–0.17 µM as estimated in the 72 h MTT assay. At the same time, tested derivatives showed lower cytotoxicity in human normal lung fibroblasts (WI-38), where IC_50_ values varied between 0.27–0.65 µM. **MM139** was the least cytotoxic to cancer cell lines, with a cytotoxicity range of 0.17–0.33 µM, while **MM137** exhibited the highest cytotoxic potential in all tested cell lines (IC_50_ values: 0.16 µM for BxPC-3, 0.11 µM for PC-3, and 0.27 µM for WI-38 cells). Apoptosis induction was the direct cause of the cytotoxic activity of the compounds, as indicated by the flow cytometry analysis with annexin V-FITC staining and acridine orange/ethidium bromide double staining. Moreover, a decrease in mitochondria membrane potential (MMP) was observed following the incubation of BxPC-3 and PC-3 cells with the tested compound, which indicated activation of the intrinsic apoptosis pathway. In silico studies suggest [19] that **MM134**, **-6**, **-7**, and **-9** compounds may act as potent and selective inhibitors of the AKT-mTOR pathway, BTK, and PD-1/PD-L1 interaction. The molecular dynamics simulation suggested that the tested compounds bind strongly and durably with molecular targets and may exert an anti-cancer effect through their inhibition [19]. Furthermore, structurally similar compounds **MM129**, **MM130**, and **MM131** exhibited cytotoxic activity with IC_50_ concentrations of 0.17–1.15 μM in four cancer cell lines (HeLa, HCT 116, PC-3, and BxPC-3) and genotoxic activity, as indicated by alkaline/neutral comet assays and γH2AX staining [34].

These facts encouraged us to explore the genotoxic activity of new **MM** derivatives (**MM134**, **-6**, **-7**, and **-9**). The tested sulfonamides exhibited genotoxic potential in BxPC-3 and PC-3 cell lines, as shown in the alkaline and neutral comet assays. Moreover, the induction of DSBs was confirmed with anti-γH2AX immunocytochemical staining. We used these assays because of the inability of alkaline and neutral comet assays to identify some kinds of DNA damage including inter- and intra-strand crosslinks induced by some agents, e.g., mitomycin. SSBs and DSBs are generated as repair intermediates during crosslink repair; however, the amount of damage formed was clearly below the detection level in both the alkaline and neutral comet assays. These lesions, on the other hand, have the potential to trigger the DNA damage response, resulting in the formation of large numbers of γH2AX foci [38].

After, 24 h incubation of cells with the tested compounds, an increase in DNA damage was observed with the concentration of compound used in the experiment, which is characteristic of genotoxic compounds. Moreover, the increase in DNA damage in the above-mentioned cells was not due to the cytotoxic activity of the compounds, as determined by the alamar blue and trypan blue viability tests. The examined compounds exhibited similar genotoxic potential in a given cell line; however, the BxPC-3 cell line was generally more prone to the DNA damaging activity of the **MM** derivatives, and 20 µM bleomycin was used in the study as a positive control. The DNA damage observed following incubation with **MM** compounds did not exceed that caused by bleomycin. However, **MM** derivatives were used in 60 to 180 times lower concentrations (for the IC_50_ values in cancer cell lines) compared to the positive control. **MM136** was the most genotoxic compound in the BxPC-3 cell line, while **MM139** exhibited the highest DNA damaging capacity in the PC-3 cell line, as estimated by the alkaline comet assay. However, neutral comet assay results indicated that in the BxPC-3 cell line, **MM134** induced the highest levels of DSBs, while PC-3 cells were more susceptible to the activity of the **MM139** compound. It is, however, difficult to compare the outcomes of the incubation with the compounds, because **MM** derivatives were used in respect of their IC_50_ values, not an arbitrarily established concentration allowing such comparison. The main objective of this study, however, was to explore whether the compounds exhibit a genotoxic effect in cancer cells. Of importance, we included the normal cell line WI-38 in the study design to examine whether the genotoxicity induced by the compounds is selective for cancer cells. Similarly to cancer cells, the WI-38 cells were treated with compounds in concentrations corresponding to the obtained IC_50_ values, which were higher (IC_50_ range: 0.27–0.65 µM) than the concentrations used to treat cancer cells (IC_50_ range: 0.11–0.33 µM). Despite that fact, the derivatives induced lower levels of DNA damage in normal cells, as indicated by the alkaline comet assay. These results only indicate potential selectivity for cancer cells and should be further examined with other cancer and normal cell lines, especially in 3D models or in vivo studies.

The induction of DSBs was confirmed with γ-H2AX staining, where the compounds induced a statistically significant increase in γ-H2AX foci formation compared to the control group. This indicated the formation of DSBs in the PC-3 cell line after 24 h incubation time with **MM** compounds. Treatment of cells with **MM134** and **MM137** induced the highest increase in γ-H2AX foci, which was somehow different from the results obtained with the neutral comet assay, where **MM139** and **MM136** exhibited the highest DSB-inducing potential. This may have resulted from discrepancies in the methodologies of both assays, the type of DNA damage they detect, and the very similar genotoxic potential of all compounds.

Our molecular docking and molecular dynamics studies indicated that the genotoxic potential of the tested compounds may result from inhibition of the CHK1 kinase involved in DDR signaling. CHK1 is a serine/threonine kinase that plays an important role in the regulation of various checkpoints within the cell cycle. These checkpoints are responsible for a variety of processes, such as the repair and stabilization of replication forks upon DNA damage. Many different CHK1 inhibitors have been developed [39]. New evidence suggests that CHK1 inhibitors may demonstrate considerable single-agent efficacy in cancers, especially those with specific DNA repair deficiencies, a constitutively active DDR, or with oncogene-induced replicative stress. This is accompanied by the increase in γ-H2AX foci formation and accumulation of DNA damage [40,41] that were observed in our study.

Prediction of the molecular targets of these compounds is crucial to understand how they affect cellular physiology. This is pivotal for the initial estimation of side effects exhibited by the compounds. Our previous studies indicated other possible targets of **MM** compounds through molecular docking and molecular dynamics approaches. These studies indicated that the apoptotic potential of the compounds may result from inhibition of the AKT-mTOR pathway, BTK kinase, and PD-1/PD-L1 interaction. This, however, needs further confirmation in in vitro and in vivo experiments. Furthermore, previous studies indicated that the compounds exhibit favorable physicochemical parameters, including drug-likeness properties, that represent an advantage for drug design purposes [19].

## 4. Materials and Methods

### 4.1. Chemicals

Trypsin-EDTA and all culture media (RPMI-1640, DMEM-F12, MEM) were purchased from Biowest (CytoGen, Poland). Agarose NMP (normal melting point), buffered saline (PBS), bleomycin sulfate, fetal bovine serum (FBS), low-melting point agarose (LMP), penicillin-streptomycin solution stabilized, sodium acetate, (NaOAc), tris(hydroxymethyl)aminomethane (TRIS), Triton X-100, Tween 20 and trypan blue were supplied by Sigma Aldrich Chemical Co. (USA). Alamar blue reagent was purchased from BioRad (USA). For γH2AX immuno-staining, bovine serum albumin (BSA) (Sigma Aldrich Chemical Co., (USA)), paraformaldehyde (PHA) (Polysciences, Inc., Warrington), fluoromount G (Invitrogen, UK), normal goat serum (Abcam), and the antibodies anti-p-H2AX Ser139 (Abcam, Cambridge, UK) and Alexa Fluor 594 goat anti-mouse (LifeTechnology, Warsaw, Poland) were used.

### 4.2. Cell Culture

BxPC-3 (pancreas adenocarcinoma, ATCC^®^ CRL-1687^TM^), PC3 (prostate cancer, ATCC^®^ CRL-1435^TM^), and WI-38 (human lung fibroblasts, ATCC^®^ CCL-75^TM^) cell lines were obtained from American Type Culture Collection (ATCC, Rockville, USA). BxPC-3 cells were grown in RPMI-1640 medium supplemented with 10% (*v*/*v*) fetal bovine serum (FBS) and 1% (*v*/*v*) of both antibiotics (streptomycin and penicillin). PC-3 cells were cultured with DMEM-F12 supplemented with 10% (*v*/*v*) FBS and 1% (*v*/*v*) of antibiotics (streptomycin and penicillin). WI-38 cells were grown in MEM supplemented with 10% (*v*/*v*) fetal bovine serum (FBS) and 1% (*v*/*v*) of both antibiotics (streptomycin and penicillin).

Cells were grown at 37 °C in a humidified atmosphere of 5% CO_2_ in the air. The culture medium was changed every 24–48 h. Subculture was performed using 0.25% trypsin/EDTA after cells reached confluence.

MycoBlue^TM^ Mycoplasma Detector kit (Vazyme biotech Co., Ltd.) was used at least every month for the control of mycoplasma contamination in the cell cultures.

The cytotoxic activity of the pyrazolo[4,3-*e*][1,2,4]triazines investigated in this study was recently published by our group [19].

### 4.3. Cell Treatment

The concentrations of the pyrazolo[4,3-*e*][1,2,4]triazine derivatives (**MM134**, **-6**, **-7**, and **-9**) used in the subsequent assays were based on previously published MTT assay results and are shown in Table 2 [19]. We decided to use these concentrations in subsequent analyses to make the workflow consistent and correlate with the previous published results.

### 4.4. Trypan Blue Staining

Trypan blue is a diazo dye that has been used for viability assessment for years. Viable cells with undamaged cellular membranes are resistant to staining and thus allow the discrimination from blue-stained dead cells with disrupted membranes [42]. Cells were seeded at a density of 1.5 × 10^5^/mL onto 12-well plates. After 24 h, cells were exposed to three concentrations of tested **MM** compounds (0.5 × IC_50_, IC_50_, and 2 × IC_50_). Cells were left for incubation for another 24 h (37 °C; 5% CO_2_). After exposure, they were transferred to Eppendorf tubes and centrifuged at 1400 rpm for 10 min at 4 °C. Afterward, the supernatant was removed, and the precipitate was diluted in PBS. Cell suspensions were mixed with 0.4% trypan blue solution in a 1:1 ratio. Approximately 300 cells were counted under a light microscope. The experiment was repeated three times. The results were represented as mean viability (%) with SD values.

### 4.5. Alamar Blue

Alamar blue works as a non-toxic and membrane-permeable redox indicator that is often used to assess the metabolic activity of cells. Over the past 50 years, the alamar blue reagent has been used to measure cell viability and cytotoxicity in a variety of cell types [43]. In this study, alamar blue (BioRAD) was used to assess cell viability before the performance of the neutral comet assay. Nunc microtiter, flat-bottomed 96-well plates were seeded at a density of approximately 1 × 10^4^ cells per 100 µL medium per well. Following the given incubation period in controlled conditions (37 °C; 5% CO_2_), cells were exposed to 11 different concentrations of tested compounds in DMSO (range 0.05–3 µM) in a volume of 100 µL medium per well. The final solvent concentration was <0.5% *v*/*v*. The experimental design included negative controls and blanks (wells without cells).

Following a 24 h incubation period, 20 µL of alamar blue was added to each well of the experimental plate. Cells were incubated in a humidified atmosphere for 4 h (37 °C; 5% CO_2_). The absorbance reading was performed at 570 and 600 nm using a spectrophotometer (microplate reader Power Wave XS BioTek Instruments, Inc., USA). The experiments were performed in triplicate.

The percentage difference between treated and control cells was calculated using the formula provided by the producer (BioRad).

(1) $$Percentage\;difference\;between\;treated\;and\;control\;cells=\frac{\left(O2\times A1\;\right)-\left(O1\times A2\right)\;}{\left(O2\times P1\right)-\left(O1\times P2\right)}\times 100\;$$

*O*1 = molar extinction coefficient (E) of oxidized alamar blue (blue) at 570 nm (*O*1 = 80,586).

*O*2 = E of oxidized alamar blue at 600 nm (*O*2 = 117,216).

*A*1 = absorbance of test wells at 570 nm; *A*2 = absorbance of test wells at 600 nm.

*P*1 = absorbance of positive growth control well (cells plus alamar blue but no test agent) at 570 nm.

*P*2 = absorbance of positive growth control well (cells plus alamar blue but no test agent) at 600 nm.

### 4.6. Comet Assays

#### 4.6.1. Alkaline Comet Assay

The comet assay is one of the most commonly used strategies utilized in the assessment of genetic toxicity caused by DNA-damaging agents. High sensitivity, simplicity, and versatility make the comet assay extremely valuable in the elucidation of possible mechanisms of genotoxicity and DNA repair at a single-cell level. The alkaline comet assay allows rapid detection of alkali-labile sites that can be easily converted into SSBs under alkali conditions (pH > 13) and the highly cytotoxic DSBs [44,45,46].

The microscopic image obtained after single-cell electrophoresis resembles a comet, where the head of the comet represents intact DNA, while the tail consists of DNA fragmented after exposure to a genotoxic agent [47].

In the alkaline comet assay, pretreated cells are usually mixed with LMP agarose and embedded on slides covered in NMP agarose [48]. Following treatment with lysis buffer allows DNA to be released from the head of the comet during electrophoresis. Commercially available software such as Open Comet or CaspLab enables easy estimation of DNA damage induced by genotoxic agents [48,49,50].

The alkaline version of the comet assay was performed according to Singh et al. (1988) [51], with modifications. BxPC-3 and PC-3 cancer cells and normal human fibroblasts (WI-38) were seeded at a density of 1.2 × 10^5^/mL onto 12-well plates. After 24 h, cells were exposed to three concentrations of tested compounds followed by IC_50_ values (0.5 × IC_50_, IC_50_, and 2 × IC_50_) or bleomycin (20 µM) used in the experiment as a positive control. The experimental design included negative controls (cells treated with DMSO at 0.4% for 24 h at 37 °C). Cells were left for incubation for another 24 h (37 °C; 5% CO_2_). After exposure, they were transferred to Eppendorf tubes and centrifuged at 1400 rpm for 10 min at 4 °C. Afterward, the supernatant was removed, and the precipitate was diluted in PBS. The next steps were performed as previously described by Kontek and Nowicka [52].

After electrophoresis (conditions: 12 V, 300 mA, 25 min), slides were stained with 1 µg/mL of the intercalating agent 4′,6-diamidino-2-phenylindole (DAPI) and coverslipped. DNA damage was assessed with a fluorescence microscope at 360 nm using CellSens (Olympus) software. A total number of approximately 50 cells per slide was chosen for further analysis.

#### 4.6.2. Neutral Comet Assay

In addition to the alkaline comet assay, we also performed the comet assay in pH conditions close to neutral pH (pH = 9) to estimate the levels of DSBs induced following 24 h incubation with the tested compounds in concentrations followed by IC_50_ values (0.5 × IC_50_, IC_50_, and 2 × IC_50_) or bleomycin (20 µM) used as a positive control in cancer cell lines (BxPC-3 and PC-3). The majority of the neutral comet assay steps were performed analogously to the alkaline version except for the electrophoresis step. In contrast to the alkaline comet assay, electrophoresis was performed in a buffer comprising 100 mM TRIS and 300 mM sodium acetate with the pH of the solution adjusted to 9.0 by glacial acetic acid, as described previously by Bukowski et al. [34]. Electrophoresis was run at 12 V, 50 mA for 60 min. Cells were washed with distilled water twice, stained with DAPI, and analyzed as described for the alkaline comet assay.

### 4.7. γH2AX Staining

PC-3 cells were seeded at a density of 3 × 10^4^/mL onto round coverslips placed on the bottom of 12-well plates. After 24 h, cells were exposed to two concentrations of the tested compounds followed by IC_50_ values (IC_50_ and 2 × IC_50_). After 24 h, cells were washed once with cold PBS and placed in 4% paraformaldehyde (PHA) in PBS for 10 min. at room temperature. After that, PHA was removed, and cells were permeabilized using 0.1% Triton X-100 for 10 min at 20 °C, followed by incubation in a blocking solution (1 mL/well) containing 2.5% BSA, 1.5% goat serum, and 0.1% Triton X-100 in PBS for 10 min at room temperature. The blocking solution was removed, and coverslips containing cells were incubated with anti-γ-H2AX primary antibodies in blocking solution (anti-gamma-H2AX (phosphor-Ser139) (Abcam, Cambridge, UK; 1:100)) for 1 h at room temperature. Cells were transferred into 12-well plates, washed twice in PBS (5 min each), transferred to plates, and incubated with Goat anti-Mouse IgG Cross-adsorbed antibody, Alexa Fluor 488 (Invitrogen; 1:500 in PBS) for 1 h at room temperature. Cells were washed twice in PBS, followed by incubation with DAPI solution (1 µg/mL) for 10 min at room temperature and two washes in PBS and distilled water. Coverslips were removed using tweezers and placed on microscope slides with a drop of Fluoromount-G mounting medium. The slides were placed flat at room temperature for 24 h and then stored at 4 °C. The slides were analyzed with a fluorescence microscope using CellSens (Olympus) software. A total number of approximately 100 cells per slide was chosen for further analysis. The experiment was performed in duplicate. In BxPC-3 cells, inefficient or non-specific staining was observed despite attempts to optimize the assay. Therefore, we discontinued further work on these cells with the assay.

### 4.8. Data Analysis

#### 4.8.1. Alkaline and Neutral Comet Assays

CASP: Comet Assay Software Project Lab (http://casplab.com (accessed on 1 January 2023)) was used to establish the median value of DNA (%) in comet tails. This parameter provides a very clear picture of what the actual appearance of the comets was like. In contrast, the tail moment is merely the product of the tail length and the tail intensity, is not linear in relation to dose, and does not provide any information regarding the comet’s physical appearance [53]. The data were presented with an interquartile range and minimal and maximal values using Graphpad Prism *7*. Statistical analysis of comet assay results was performed using Statistica software. The Kruskal–Wallis test was used to show a statistically significant difference between groups. Multiple comparisons using mean ranks for all groups module of Statistica software were used. A *p*-value less than 0.05 was considered statistically significant. In all groups, *N* > 200.

#### 4.8.2. γH2AX Staining

Images for green γ-H2AX (Alexa Fluor 488) and blue nuclear signals (DAPI) were taken using an Olympus camera. ImageJ software “find maxima” and “count” functions were used to determine the number of γ-H2AX foci in each cell. The “preview point selection” option and “prominence” were chosen based on the accuracy of coverage of γ-H2AX foci. Data were analyzed using GraphPad Prism 7.0 software system (GraphPad Prism Software Inc., USA) and Statistica software. The Kruskal–Wallis test was used to show a statistically significant difference between groups. Multiple comparisons using mean ranks for all groups module of Statistica software were used. A *p*-value less than 0.05 was considered statistically significant (*p* < 0.05). In all groups, *N* > 200.

### 4.9. Computational Studies

#### 4.9.1. Molecular Docking

The atomic coordinates of the investigated factors belonging to the DDR pathway were obtained from the PDB databank (http://www.rcsb.org/ (accessed on 1 January 2023)). Macromolecules were prepared for docking through the assignment of autodock atom type (AD4), the addition of Gasteiger charge, and its equal distribution among the macromolecular residues followed by saving them in the default autodock format PDBQT using AutoDock Tools (The Scripps Research Institute, La Jolla, CA, USA).

The structures of **MM** ligands (**MM134**, **-6**, **-7**, and **-9**) were drawn using ChemDraw 8.0 software and converted into three-dimensional structures with subsequent energy minimization using the MM2 force field. The ligands were prepared for molecular docking simulation through the detection of aromatic carbons and rotatable bonds, setting of automatic torsion number, margin non-polar hydrogens, and addition of Gasteiger charges using AutoDock Tools.

For each of the investigated DDR proteins, a grid box was constructed that was adequate for covering all of the extended conformations of the complexed reference ligands and the majority of the macromolecular residues that were involved in the interactions (Table 3). The grid parameters for each of the targets were saved in a respective grid parameter file (GPF) for each DDR factor. This was performed using the Autogrid utility of the Autodock suite to further generate the map files that are necessary for carrying out molecular docking simulations. The dockings of reference ligands (that were already co-crystalized with PDB macromolecule targets) with the macromolecules were performed alongside the docking of **MM** compounds.

#### 4.9.2. Molecular Dynamics

The molecular dynamics simulations of **MM137** with APE1, CHK1, and TOPO1 (complexes exhibiting highest binding affinities) were performed using the Desmond software with an OPLS force field at the time frame of 100 nanoseconds (ns) at constant temperature and pressure conditions, as previously described in NVT condition. Analyses of trajectories were performed using Desmond utilities to evaluate root-mean-square deviation (RMSD), root-mean-square fluctuation (RMSF), the radius of gyration, and interacting bonds.

## 5. Conclusions

The present findings showed diverse cellular responses to the tested pyrazolo[4,3-*e*]tetrazolo[1,5-*b*][1,2,4]triazine sulfonamides, which were specific for the cell line and dependent on the compound used in the experiment. Genotoxicity testing confirmed the high antineoplastic properties of **MM134**, **-6**, **-7**, and **9**. Moreover, these effects were restricted mainly to cancer cells, indicating selectivity in targeting malignant cells. The DNA damaging potential of the compounds may be associated with CHK1 kinase inhibition, as suggested by in silico investigations. However, this needs confirmation in further studies.

## Figures and Tables

**Table 1:** Molecular docking results: the results of molecular docking studies of **MM** compounds and reference ligands against macromolecular structures of proteins involved in the DDR pathway. The common binding residues for MM compounds and reference ligands are bolded.

MM Compound	Binding Energy (Kcal/mol)	Binding Residues	Reference Ligand	Binding Energy (Kcal/mol)	Binding Residues
APE-1 DNA-(apurinic or apyrimidinic site) endonuclease 6MKO					
**MM134**	−9.18	**Asp308**, **His309**, Gly176, Gly178, Arg177, Ala230, Trp280, Asn174	Methoxyamine (No native ligand was complexed)	−5.74	Asn212, Asp210, His309, Asn68, Asp308, Glu96
**MM136**	−8.07	Gly178, Arg177, Gly176, **Asp308**, Trp280, **His309**, Leu282, Phe266			
**MM137**	−9.64	**Asp308**, **His309**, Gly176, Ala230, Trp280, Phe266, Asn212, Tyr171			
**MM139**	−7.63	Tyr269, Phe266, Met270, Thr268, Ala230, Asn174, Gly231			
ATR Serine/threonine-protein kinase ATR 4WAF					
**MM134**	−10.06	Asp933, Ile848, Tyr836, **Ile932**, **Val850**, **Met922**, Trp780, Thr856, His855	N,N-dimethyl-4-[(6R)-6-methyl-5-(1H-pyrrolo[2,3-b]pyridin-4-yl)-4,5,6,7-tetrahydropyrazolo[1,5-a]pyrazin-3-yl]benzenesulfonamide	−10.62	Ile848, **Ile932**, **Val850**, Val851, **Met922**, Glu849, Thr856, His855, Gln859, Lys802, Met800
**MM136**	−10.32	Tyr836, **Ile932**, **Val850**, Arg852, Val851, **Met922**, Asn853, Ser854, Trp780			
**MM137**	−10.95	Tyr836, Val851, **Met922**, **Val850**, **Ile932**, Trp780, Ser854, Arg852			
**MM139**	−10.34	Ile848, **Ile932**, **Val850**, Trp780, Thr856, His855			
ATM Serine/threonine-protein kinase ATR 7NI5					
**MM134**	−9.02	Pro2775, Gln2874, Trp2769, **Leu2767**, Leu2715, Ile2888, **Tyr2755**	KU-55933	−10.58	**Tyr2755**, **Cys2770**, Glu2768, **Leu2767**, Lys2717, Trp2769, Leu2715, Pro2699, Ala2693, Pro2775, Leu2877
**MM136**	−9.17	Asp2720, Lys2717, Leu2877, Trp2769, Thr2773, **Cys2770**, **Tyr2755**, **Leu2767**, Ile2888, Asn2697			
**MM137**	−9.16	Ala2693, Glu2778, Ile2888, Lys2717, **Tyr2755**, **Leu2767**, Cys2770, Trp2769, Leu2877, Pro2775, Val2774			
**MM139**	−10.05	Trp2769, Leu2877, **Tyr2755**, Ile2888, Gly2694, Pro2699, Leu2715, **Cys2770**			
CHK1 Serine/threonine-protein kinase Chk1 2YM8					
**MM134**	−9.75	**Cys87**, **Ala36**, **Leu137**, Leu15, **Val23**, **Leu84**, **Lys38**, Phe149	(R)-5-(8-chloroisoquinolin-3-ylamino)-3-(1-(dimethylamino)propan-2-yloxy)pyrazine-2-carbonitrile	−9.14	**Ala36**, **Leu84**, Glu85, Val68, **Lys38**, Glu134, Asn135, **Leu137**, **Val23**, **Cys87**, Tyr86, Leu15
**MM136**	−10.44	**Cys87**, Tyr86, **Leu137**, **Leu84**, **Ala36**, Val68, Phe149, Lys38, **Val23**, Gly90			
**MM137**	−10–82	**Cys87**, **Ala36**, **Leu137**, **Val23**, Val68, **Lys38**, **Leu84**, Phe149, Asn59, Tyr86			
**MM139**	−10.61	Cys87, Phe149, **Lys38**, **Val23**, **Ala36**, **Leu137**, Leu15			
CHK2 Serine/threonine-protein kinase Chk2 2W0J					
**MM134**	−9.72	Leu301, **Lys249**, Asn352, Ile299, Glu308, **Val234**, Leu354, **Leu226**	4,4′-diacetyldiphenylurea-bis(guanyl-hydrazone)	−9.76	Glu273, Thr367, Ile299, **Lys249**, Leu301, **Val234**, Leu303, **Leu226**, Glu305
**MM136**	−9.64	**Leu226**, Met304, Ala247, Leu301, Ile299, **Lys249**, Thr367, Leu354, **Val234**			
**MM137**	−9.72	**Leu226**, Met304, Ala247, **Val234**, Leu354, **Lys249**, Leu301, Ile299			
**MM139**	−10.23	Phe369, **Lys249**, Ala247, Leu354, **Leu226**, Met304, Leu303, **Val234**, Thr367			
PARP-1 Poly [ADP-ribose] polymerase 1 7ONS					
**MM134**	−10.51	Glu763, Tyr889, Asp766, Ala880, Leu877, Arg878, Leu769, Pro881, Tyr896	7-[[4-(1,5-dimethylimidazol-2-yl)piperazin-1-yl]methyl]-3-ethyl-1~{H}-quinolin-2-one	−10.36	Gly863, **Arg878**, Leu769, Pro881, Asp770, Asn767, Tyr896, **Tyr907**, Lys903, **His862**, Ala898
**MM136**	−9.88	**Tyr907**, **His862**, Asp766, Asp770, Leu877, Ile872, Arg878, Ala880			
**MM137**	−10.29	Arg878, Ala898, **Tyr907**, Tyr896, **His862**, Ile879			
**MM139**	−10.36	Ala880, Leu769, Pro881, Arg878, Asn767, Arg865, His909, **Tyr907**, **His862**, Asp770			
RPA70 Replication protein A 70 kDa DNA-binding subunit 4IJL					
**MM134**	−5.81	Arg41, **Ile95**, Ile83	{[5-(3-chloro-1-benzothiophen-2-yl)-4-phenyl-4H-1,2,4-triazol-3-yl]sulfanyl}acetic acid	−5.97	Leu87, Met57, Arg41, Ala59, Thr60, **Ile95**, Val93, Asn85
**MM136**	−6.16	Met97, Ala59, Gln61, Ile83, **Ile95**			
**MM137**	−5.71	Arg41, Met92, Ala59, **Ile95**, Met57, Ile83			
**MM139**	−7.08	Met57, **Ile95**, Ile83			
TOP1 Topoisomerase I 1TL8					
**MM134**	−9.81	Met428, Asn352, Lys436, **Arg364**, Lys425, Thrr426, **DA13**, DA14	2,3-dimethoxy-12H-[1,3]dioxolo[5,6]indeno[1,2]isoquinolin-6-ium	−9.53	Asn722, Thr718, **Arg364**, DT10, DG12, **DA13**
**MM136**	−10.44	Lys425, **Arg364**, Glu356, Asn722, DT10, DG12, **DA13**			
**MM137**	−10.69	Lys425, Lys374, **Arg364**, DT10, DG12, **DA13**			
**MM139**	−10.34	Lys532, DT10			
TOP2B Topoisomerase II 3QX3					
**MM134**	−8.54	Glu477, Gly478, DC8, **DA12**, **DG13**	Etoposide	−9.98	Asp479, Arg503, Gly478, Met782, DC8, **DA12**, **DG13**
**MM136**	−8.24	Arg503, Ala779, Met782, **DA12**, **DG13**			
**MM137**	−8.32	Ser480, Leu502, Asp559, Asp557			
**MM139**	−7.76	Gly478, Arg503, DC8, **DA12**, **DG13**			
WEE1 Wee1-like protein kinase 2IN6					
**MM134**	−9.14	Asp386, **Ile305**, **Cys379**, **Phe433**, **Val313**, **Ala326**, Ser307	PD311839	−9.45	Gly382, **Cys379**, **Phe433**, **Ala326**, Glu377, **Val313**, Val360, Asn376, **Lys328**, **Ile305**, Gly306, Ser307
**MM136**	−9.99	**Cys379**, Asn376, Ile374, His350, Gly382, **Val313**, **Ala326**, **Phe433**, Val360, **Lys328**, Asp463			
**MM137**	−10.07	**Ile305**, **Val313**, **Lys328**, Asp463, Ile374, Asn376, **Phe433**, Val360, **Ala326**, **Cys379**			
**MM139**	−9.92	**Lys328**, **Val313**, **Ala326**, **Phe433**, **Ile305**, Tyr378, Gly382, Ser383			

**Table 2:** MTT results after 72 h: mean IC_50_ values ± SD obtained after 72 h incubation of cells with the tested **MM** compounds (**MM134**, **-6**, **-7** and **-9**) using MTT assay.

Compound	Cell Line		
	IC_50_ Value ± SD		
	BxPC-3	PC-3	WI-28
**MM134**	0.32 ± 0.1	0.16 ± 0.02	0.65 ± 0.07
**MM136**	0.25 ± 0.08	0.13 ± 0.01	0.48 ± 0.09
**MM137**	0.16 ± 0.04	0.11 ± 0.007	0.27 ± 0.04
**MM139**	0.33 ± 0.14	0.17 ± 0.003	0.64 ± 0.06

**Table 3:** Grid box coordinates used in molecular docking studies: the coordinates of the grid box for all of the macromolecular targets belonging to the DDR pathway used in the current study.

Target	Full Name	PDB Code	x-D	y-D	z-D	Spacing (Ả)	X-Center	Y-Center	Z-Center
APE-1	DNA-(apurinic or apyrimidinic site) endonuclease	6MKO	50	50	50	0.381	20.283	22.147	20.508
ATR	Serine/threonine-protein kinase ATR	4WAF	40	40	46	0.397	−1.215	8.294	−17.439
ATM	Serine/threonine-protein kinase ATR	7NI5	40	40	40	0.397	111.559	150.337	210.394
CHK1	Serine/threonine-protein kinase Chk1	2YM8	40	40	40	0.414	15.394	−1.219	11.745
CHK2	Serine/threonine-protein kinase Chk2	2W0J	40	40	46	0.408	37.373	−31.962	9.087
PARP-1	Poly [ADP-ribose] polymerase 1	7ONS	40	40	46	0.397	10.962	42.743	7.819
RPA70	Replication protein A 70 kDa DNA-binding subunit	4IJL	40	40	46	0.369	−5.944	−9.334	1.963
TOP1	Topoisomerase I	1TL8	40	40	40	0.392	22.245	−4.32	27.329
TOP2B	Topoisomerase II	3QX3	40	40	40	0.553	32.884	95.413	50.785
WEE1	Wee1-like protein kinase	2IN6	40	40	40	0.408	3.987	52.579	26.054

## Data Availability

The data presented in this study are available in the main text of this article or on request from the corresponding author.

## Abbreviations

ABL	ABL protein kinase
AKT	RAC-alpha serine/threonine-protein kinase
ATM	serine/threonine protein kinase ATM
ATR	serine/threonine protein kinase ATR
BAX/BAK	pro-apoptotic protein BAX/BAK
BSA	bovine serum albumin
BTK	Bruton’s tyrosine kinase
CA	carbonic anhydrase
CDK	cyclin-dependent kinase
CHK1	serine/threonine protein kinase CHK1
DAPI	4′,6-diamidino-2-phenylindole
DDR	DNA damage response
DSB	double-strand breaks
FBS	fetal bovine serum
IRF1	Interferon regulatory factor 1
JAK	tyrosine-protein kinase JAK
MMP	mitochondria membrane potential
mTOR	mammalian target of rapamycin
NMP	normal melting point agarose
PARP1	poly[ADP-ribose] polymerase 1
PBS	buffered saline
PD-1	programmed death 1
PD-L1	programmed death ligand 1
PHA	paraformaldehyde
PI3K	phosphatidylinositol-4,5-bisphosphate 3-kinase
s-ICAM-1	soluble intercellular adhesion molecule-1
SSBs	single-strand breaks
STAT1/3	signal transducer and activator of transcription 1/3
TP53	cellular tumor antigen p53
TRIS	tris(hydroxymethyl)aminomethane
